# Clinical Empathy and Its Correlates Among Indian Medical Students: A Cross-Sectional Study of Bihar

**DOI:** 10.7759/cureus.40159

**Published:** 2023-06-08

**Authors:** Kritika Tiwari, Neeraj Agarwal, Sanjay Pandey

**Affiliations:** 1 Community Medicine, Army College of Medical Sciences, New Delhi, IND; 2 Community and Family Medicine, All India Institute of Medical Sciences, Bibinagar, Bibinagar, IND; 3 Community and Family Medicine, All India Institute of Medical Sciences, Patna, Patna, IND

**Keywords:** doctor-patient relationship, medical curriculum, aetcom, jefferson scale for physician empathy - student version, medical students, clinical empathy

## Abstract

Background

Clinical empathy is standing in the patient’s shoes and perceiving his/her emotions to experience the patient’s feelings. Practicing empathy ensures an enticing prospect in patient care. This study was done among undergraduate medical students to assess their empathy level and the factors affecting it.

Methods

This was a cross-sectional study conducted with 400 medical students in Bihar, India. Students not willing to participate were excluded from the study. The coding system was designed to strictly maintain anonymity. The study tools included the Jefferson Scale for Physician Empathy - Student Version (JSPES), a semi-structured questionnaire on the general profile, a perceived stress scale (PSS), and a multidimensional scale of perceived social support (MSPSS). Participants were allotted 20 minutes to complete the test and submit their responses. Results were expressed as means and standard deviations (SDs), with appropriate statistical tests applied. The data were presented in tables, and statistical significance was checked at a 5% level. All statistical analyses were conducted using SPSS software.

Results

The arithmetic mean (±SD) of empathy scores was 99.87±14.71. Empathy was found to be positively correlated with social support and negatively with stress. The factors found to be significantly associated with empathy on univariate analysis were subjected to stepwise multiple linear regression, which provided a six-factor model comprising gender, choice of future specialty, stress, social support, residence, substance abuse, and stay in hospital as an attendant.

Conclusions

Stress and social support were found to be significant predictors of empathy. The female gender, living in urban areas, and having previous experience of hospital stay as an attendant of a patient were positively associated with empathy. In contrast, choosing a technical branch as a future specialty and substance abuse were negatively associated with empathy. Stress management, enhancement of social support, and avoidance of habit-forming substances could be beneficial in improving empathy among doctors. Since we could only identify a few factors, we recommend further studies on this topic to explore other factors.

## Introduction

Empathy, derived from the Greek word 'Empatheia', has recently gained attention in various fields, including medicine [1]. Clinical empathy is studied across four dimensions - cognitive, emotive, moral, and behavioral - contrasting with sympathy, which predominantly embodies the emotive dimension [2]. Cultivating empathy as a personality attribute in doctors has been shown to enhance clinical performance and patient care [3]. However, teaching empathy remains a grey area in the Indian medical curriculum. With the introduction of the Attitude, Ethics, and Communication (AETCOM) module in the new MBBS syllabus in India [4], studies measuring empathy prior to this reform have become cardinal tools for assessing the module's future impact on the empathy levels of medical students. This study aimed to assess empathy levels among medical students and explore the factors affecting these empathy scores.

## Materials and methods

This was a cross-sectional study conducted among medical students from a medical college in Bihar, India, encompassing MBBS students from the 1st to 9th semesters, as well as interns. Permission was obtained from the institute's ethical committee and the authors of the Jefferson Scale for Physician Empathy - Student Version (JSPES) prior to the inception of the study. Assuming a standard deviation (SD) of the empathy score as 12.9 from a previous study [5], a tolerable error of 1.5% at a 95% confidence level, and a non-response rate of 10%, the minimum sample size was calculated to be 313 using the formula 1.962σ2/l2 (σ = standard deviation, l = tolerable error). All students present in their respective classes on the day of the study were included after obtaining informed consent, which brought the final sample size to 400. Participants were given 20 minutes to complete and submit their responses to the test.

Clinical empathy was measured using the JSPES, a validated 20-statement scale that utilizes a 7-point Likert system (with a maximum possible score of 140). A higher score on this scale indicates a greater level of empathy. Negatively worded items were reverse-coded for analysis in accordance with the scale's guidelines. Additional study tools included a semi-structured questionnaire for the general profile, a Perceived Stress Scale (PSS), and a Multidimensional Scale of Perceived Social Support (MSPSS). The PSS is a self-rating tool designed to gauge perceived stress over the past month [6]. It consists of 10 items on a 5-point Likert scale (maximum score: 40), with higher scores signifying greater perceived stress. The MSPSS assesses perceived social support from family, friends, and significant others [7]. It contains 12 items on a 7-point Likert scale (maximum score: 84), with higher scores, indicating higher levels of perceived social support. Data entry and analysis were done by SPSS software (version 22). Mean and SDs were calculated for JSPES, PSS, and MSPSS scores. The internal consistency of these scales was measured by calculating Cronbach's alpha. Data normality was tested by P-P plot. Pearson's correlation coefficient was calculated for JSPES, PSS, and MSPSS. Statistical tests applied were independent samples t-test and one-way ANOVA followed by a posthoc test. Multiple regression analysis was done to arrive at the final model of predictor variables of empathy. The significance level was kept at 5%.

## Results

Table 1 shows the general profile of the study population comprising of gender, age (classified according to JSPES age categories), semester of MBBS, future choice of specialty, residence, substance abuse, and experience of a hospital stay as an attendant with any ill relative/friend.

**Table 1:** General profile of study population.

	Number (N = 400)	Percentage
Gender		
Male	243	60.8
Female	157	39.3
Age (years)		
<22	272	68
22-24	120	30
25-27	8	2
MBBS semester		
1^st^	47	11.8
3^rd^	85	21.3
5^th^	94	23.5
7^th^	87	21.8
9^th^	61	15.3
Intern	26	6.5
Choice of speciality		
Medical	157	39.3
Surgical	134	33.5
Technical	27	6.8
Undecided	82	20.2
Residence		
Rural	118	29.5
Urban	282	70.5
Substance use		
Never	345	86.3
Occasionally	46	11.5
Regularly	9	2.3
Stay in hospital with ill relative/friend		
Yes	196	49.0
No	204	51.0

The means and SDs were calculated for the three scales used, i.e., JSPES, PSS, and MSPSS (as shown in Table 2). The mean and SD of empathy scores were calculated to be 99.87 ± 14.7, with maximum and minimum values of 47 and 134, respectively.

**Table 2:** Summary statistics and reliability coefficient of JSPES, PSS and MSPSS among the study population MSPSS: Multidimensional Scale of Perceived Social Support; PSS: Perceived Stress Scale; JSPES: The Jefferson Scale of Physician Empathy - Student Version.

	JSPES	PSS	MSPSS
Mean (±SD)	99.87 ± 14.7	19.71 ± 6.7	62.17 ± 13.9
Cronbach’s alpha (α)	0.82	0.83	0.90

The reliability of these scales for our study population was measured by Cronbach’s alpha, which showed good internal consistency for all the scales (α>0.8). The P-P plot showed that empathy scores were normally distributed among the study population.

Empathy scores showed significant correlations with stress scores and social support scores. The correlation of JSPES was weakly negative with PSS and weakly positive with MSPSS (Table 3).

**Table 3:** Correlation of JSPES with PSS and MSPSS. MSPSS: Multidimensional Scale of Perceived Social Support; PSS: Perceived Stress Scale; JSPES: The Jefferson Scale of Physician Empathy - Student Version.

	PSS		MSPSS	
	r*	p	r*	P-value
JSPES	-0.12	0.02	0.22	<0.001
*Pearson’s correlation coefficient				

The correlates of empathy scores among the study population are depicted in Tables 4 and 5. The association of clinical empathy with gender, residence, and hospital stay was analyzed using an independent samples t-test, while the associations with future choice of specialty and substance abuse were analyzed using a one-way ANOVA. Levene's test was applied to check for the homogeneity of variance. It was found that females had significantly higher empathy scores compared to males. A similar result was observed for students belonging to urban families as compared to those from rural ones. Empathy was significantly higher in those with an experience of staying in a hospital as an attendant with an ill relative or friend. One-way ANOVA followed by Scheffe's post hoc test showed that students planning to opt for technical branches (e.g., microbiology, pathology, radiology, etc.) had significantly lower empathy scores compared to those who wanted to choose medical or surgical branches or were still undecided. Similarly, one-way ANOVA followed by Scheffe's post hoc test showed that students who had never used any habit-forming substance had higher empathy scores compared to both occasional and regular users of such substances.

**Table 4:** Association of empathy with gender, residence, and hospital stay among the study population.

Variables	Mean (±SD)	t statistic	P-value*
Gender			
Male	97.2 (±14.1)	-4.6	<0.001
Female	104.0 (±14.6)		
Residence			
Rural	96.0 (±14.5)	-3.4	0.001
Urban	101.5 (±14.5)		
Stay in hospital with ill relative/friend			
Yes	101.1 (±15.1)	1.7	0.04
No	98.6 (±14.2)		
*Equal variances assumed (Levene’s test p value>0.05), independent samples t-test			

**Table 5:** Association of empathy with choice of specialty and substance use.

Variables	Mean (±SD)	F statistic	P-value*	Post hoc test^#^
Choice of speciality				
Medical	99.76 (±15.4)	2.88	0.036	p<0.05 between technical and other variables
Surgical	100.7 (±13.6)			
Technical	92.2 (±16.1)			
Undecided	101.2 (±14.0)			
Substance use				
Never	100.8 (±14.1)	6.09	0.002	p<0.05 between never and other variables
Occasionally	94.9 (±15.9)			
Regularly	88.8 (±21.6)			
*Equal variances assumed (Levene’s test p value>0.05), One-way ANOVA ^#^ Scheffe’s post hoc test				

The variables that were found to be significantly associated with clinical empathy scores on univariate analysis were subjected to multiple regression analysis by the stepwise method. Table 6 shows the values of R squared, β coefficient, and p values obtained by the stepwise multiple regression of JSPES scores on predictor variables. The regression model consisted of six factors (gender, residence, substance use, hospital stay as attendant, PSS, and MSPSS). This model was found to have a high statistical significance by ANOVA and was found to explain 15.7% of the variability in empathy scores. The positive predictors of empathy in the model were female gender, urban residence, social support, and stay in the hospital as an attendant. In contrast, the negative predictors were stress and substance abuse.

**Table 6:** Multiple regression analysis of JSPES scores on predictor variables. MSPSS: Multidimensional Scale of Perceived Social Support; PSS: Perceived Stress Scale; JSPES: The Jefferson Scale of Physician Empathy - Student Version.

Predictors variables*	β coefficient^@^	P-value	R^2 #^
Gender	0.2	0.001	0.157
Residence	0.1	0.003	
Substance use	-0.1	0.003	
Hospital stay as attendant	0.1	0.043	
PSS	-0.2	0.004	
MSPSS	0.2	<0.001	
*Stepwise multiple regression analysis, ^@^ β coefficient : regression coefficient ^#^ R^2^: Coefficient of multiple determination, Model p value <0.001			

## Discussion

This research study, involving 400 Indian medical students, was conducted to assess empathy and its correlates using JSPES. The mean empathy score derived from JSPES was 99.87 ± 14.7, which aligns with findings from some Indian studies conducted in North India at Dehradun [5], Western India at Nagpur [8], and South India at Karnataka [9]. Another Indian study done in New Delhi [10] and a study from Nepal [11] have reported slightly lower empathy scores, while a study in Iran [12] reported a significantly lower mean empathy score of 61.11 ± 2.31. An Indian study conducted at Vijayawada, which included both postgraduate and undergraduate students, reported higher empathy scores [13]. Similarly, a study performed in Australia, with students from different ethnicities, showed a higher mean empathy score than our study [14]. Likewise, studies conducted in other foreign countries, such as Korea [15], Brazil [16], and South Africa [17], have reported higher scores. These differences could potentially be attributed to cultural diversity and variations in teaching syllabi.

Like various other studies, this study also showed that females were more empathetic than males [8, 10, 12, 14, 15, 18-23]. Some studies have tried to explain the gender relationships of empathy, which includes studies on emotional receptivity and understanding, as well as studies hypothesizing its genetic basis and neurological basis [16, 17, 24, 25]. However, some studies have shown no such association between gender and empathy [11, 26-28]. This study could not appreciate any association between empathy and age. Similarly, this study did not show any association between empathy and MBBS semester. These domains have shown incongruent results in various studies. Results similar to this study have been found in other studies as well [8, 10, 14]. A study performed in Kolkata showed an inverse relation between age and empathy [18]. An inverse relationship has also been found between empathy and clinical exposure in some studies [ 12, 18, 24, 29]. A study performed at Dehradun has shown that empathy increases at the beginning of clinical exposure but later decreases as the clinical exposure increases [5]. A study by Biswas B et al. [18] has shown that empathy improved among interns, but our study could not find such an association. Some other studies have found no association between empathy and choice of future specialty [10, 25]. In contrast, students who preferred people-oriented branches were found to be more empathetic than those preferring technical branches in some studies [18, 24, 22]. This study also found that the students who wanted to opt for technical branches had significantly lower empathy scores. However, it was not found to be a significant factor in multivariate analysis.

A study performed at Kolkata by Biswas B et al. [18] has concluded that students from rural backgrounds were more empathetic, but in this study, we found that those from urban backgrounds were more empathetic. On regression analysis, we found a six-factor model consisting of the female gender, social support, hospital stay as an attendant, and urban background as the positive correlates, whereas stress and substance abuse as the negative correlates of empathy. The regression model of the Kolkata study by Biswas B et al. consisted of the female gender, MBBS semester, rural residence, career satisfaction, and future career choice [18]. Career satisfaction was found to be a significant predictor of empathy in our study, too, on univariate analysis, but it was removed in the regression analysis. Similar to our study, stress was found to be negatively correlated, and social support positively correlated with empathy in a study by Biswas B et al. [18].

Limitations of the study

It was a small-scale study conducted in one medical college, and we could identify only a few correlates of clinical empathy.

## Conclusions

Clinical empathy is a multidimensional concept. We got a six-factor model based on regression analysis. Empathy showed a positive association with female gender, social support, hospital stay, and urban residence, whereas a negative association with stress and substance abuse. Hence, stress management, amelioration of social support, and refraining from using any habit-forming substance can help increase empathy among medical students. More research studies are recommended to identify other correlates of clinical empathy. Further research after the introduction of the AETCOM module is also recommended.
